# Celery (*Apium graveolens*) as a potential antibacterial agent and its effect on cytokeratin-17 and other healing promoters in skin wounds infected with methicillin-resistant *Staphylococcus aureus*

**DOI:** 10.14202/vetworld.2020.865-871

**Published:** 2020-05-09

**Authors:** Yos Adi Prakoso, Chylen Setiyo Rini, Asih Rahayu, Miarsono Sigit, Dyah Widhowati

**Affiliations:** 1 Faculty of Veterinary Medicine, University of Wijaya Kusuma Surabaya, East Java, 60225, Indonesia; 2 Technology of Medical Laboratory, Faculty of Health, University of Muhammadiyah Sidoarjo, East Java, Indonesia, 61261, Indonesia

**Keywords:** antimicrobial, celery, cytokeratin-17, methicillin-resistant *Staphylococcus aureus*, wound healing

## Abstract

**Background and Aim:**

Antimicrobial resistance is a global problem caused by extensive utilization of antibiotics that promote gene resistant among bacteria, including *Staphylococcus aureus*. This study aimed to analyze the potential effects of celery (*Apium graveolens*) extract as an antioxidant and antimicrobial agent against methicillin-resistant *S. aureus* (MRSA), *in vitro* and *in vivo*.

**Materials and Methods:**

Celery was extracted and tested against a MRSA isolate *in vitro*. The minimum inhibitory concentration (MIC) against MRSA for the celery extract (CE) was determined to be 0.1% and it was formulated into a cream. A total of 30 female *Sprague Dawley* rats were divided into five groups: Group 1, negative control; Group 2, positive control; Group 3, treated with 0.05% CE cream; Group 4, 0.1% CE cream; and Group 5, 0.2% CE cream. All animals in the groups were exposed to a full-thickness skin biopsy on the dorsal portion, and they were infected with 30 µL of 10^5^ colony-forming units of the MRSA isolate. The treatment was administered twice a day for 7 days. The skin samples were collected on days 3 and 7 after the treatment. The skin tissue was examined histologically using hematoxylin and eosin, Gram staining, and immunohistochemistry against cytokeratin (CK)-17.

**Results:**

Results showed that 0.2% of CE cream was the best treatment for wounds infected with MRSA. CE (0.2%) cream increased skin reepithelialization, fibroblast proliferation, and CK-17 expression; it also decreased the percentage of wound area, inflammatory cell infiltration, and bacterial colonization in skin wound tissue compared to the other treatments (p≤0.05).

**Conclusion:**

This study demonstrated that celery could be utilized as an alternative herbal therapy against MRSA-associated skin infections.

## Introduction

Antimicrobial resistance is a global problem caused by extensive utilization of antibiotics. The uncontrolled utilization of antibiotics in society promotes gene resistance among the bacteria [1]. One of the successive bacterial strains resistant to several derivatives of antibiotics is *Staphylococcus aureus* and this is known as methicillin-resistant *S. aureus* (MRSA). MRSA can cause severe soft-tissue infections, including skin infections [2], and an increase in the risk of amputation in chronic ulcers [3]. MRSA is transmitted into the skin through open wounds and causes septicemia due to further development of infections [4]. Therefore, the utilization of antibiotics must be done carefully and under medical expertise to prevent an increase in the MRSA.

In the past decade, natural antioxidants have been widely developed because they not only promote tissue repair but also have potential as antimicrobial agents. Celery (*Apium graveolens*) is commonly used as food, but it could also be used as a source of exogenous natural antioxidant [5]. Celery extract (CE) contains flavonoids and phenolic compounds that not only inhibit reactive oxygen species (ROS) during inflammation [6] but also restrain bacterial colonization [7]. The management of MRSA colonization using exogenous antioxidants promotes faster healing in the skin wound through the activation of anti-inflammatory mechanisms and cell activation.

One of the prominent cells present during skin wound healing is the keratinocytes [8]. Keratinocytes express cytokeratins (CKs) that promote cell migration, proliferation, and adhesion [9]. The most significant CK is CK-17. CK-17 promotes the survival of keratinocytes, which increases reepithelialization to maintain the skin barrier [10]. In recent years, excellent barrier formation through the modulation of CK-17 expression maintains skin hydration and fluidity, which stabilizes tissue matrix components and improves remodeling [11].

This study aimed to analyze the potential role of CE in the expression of CK-17 and its involvement in promoting skin wound healing during MRSA infection.

## Materials and Methods

### Ethical approval

The Ethical Clearance Committee of the Faculty of Dental Medicine, University of Airlangga, East Java, Indonesia, approved all procedures involving animals in this study with the following registration number: 313/HRECC.FODM/VI/2019.

### Study period and study location

The study was conducted from April until November 2019. All experimental procedures were performed in the Integrated Laboratory, Faculty of Health, University of Muhammadiyah Sidoarjo, East Java, Indonesia.

### Herbal extraction

Celery was collected from a local herbal market in Sidoarjo, East Java, Indonesia. The Indonesian Institute of Sciences, Purwodadi, East Java, Indonesia, identified and authenticated the species of the collected celery to be *A. graveolens*. The celery was dried at 80°C in an oven for an hour. The components of dried celery were extracted using 70% methanol, and the extract was refrigerated until use.

### Preliminary study

The CE was tested against the MRSA isolate obtained from a clinical specimen that was taken from the Department of Clinical Microbiology, Faculty of Medicine, University of Airlangga, East Java, Indonesia. The efficacy of the CEs was tested against MRSA using disk diffusion methods and was followed by the determination of the minimum inhibitory concentration (MIC) [12]. The disk diffusion test was expressed as the percentage inhibition of diameter growth (PIDG). There was a larger PIDG observed with higher concentrations of the CE. Further, the MIC test demonstrated that 0.1% of the concentration optimally inhibited the growth of MRSA by more than 50% of the total bacteria based on the absorbance value of MRSA suspension. Based on the minimum value of the standard deviation of PIDG and MIC, the CE has a consistent potency against MRSA both in solid and broth media (Table-1).

**Table-1:** The PIDG and MIC.

PIDG (%)±SD		Minimum inhibitory concentration (%)±SD	
0	0.00±0.00	0	3.54±0.01
10	17.79±0.47	0.05	1.85±0.00
20	96.56±0.39	0.1	0.86±0.01*
30	207.89±1.12	0.2	0.54±0.01**
40	331.12±0.57	0.4	0.34±0.11**
50	488.22±0.67	0.8	0.24±0.00**
60	677.30±1.78**	1.6	0.15±0.04**
70	914.85±0.94**	3.2	0.07±0.00**
80	1188.33±1.07**	6.4	0.02±0.00**
90	1300.75±0.68**	*Minimum optimal concentration	
100	1568.99±1.13**	**Higher optimal concentration	

PIDG=Percentage inhibition of diameter growth, SD=Standard deviation

### Cream formulation

Based on the results of the preliminary study, 0.1% was recognized as the MIC against the MRSA isolates *in vitro*. This concentration was then formulated into a cream base formulation [13].

### Animal models and research design

Female *Sprague Dawley* rats weighing 200-250 g were obtained from the Laboratory of Animal Models, Faculty of Health, University of Muhammadiyah Sidoarjo, East Java, Indonesia. There were a total of 30 rats used for the study. The rats were divided into the following five groups: Group 1 was a negative control (without treatment); Group 2 was a positive control and treated with a cream base without the extract; Group 3 was treated with 0.05% CE cream; Group 4 was treated with 0.1% CE cream; and Group 5 with 0.2% CE cream. All the animals in each group were shaved and subsequently two consecutive 6 mm thick skin biopsies were taken from the dorsal side of the rats, under anesthesia, using a combination of ketamine (50 mg/kg) and xylazine (4 mg/kg). Following this, wounds were artificially infected using 30 µL of 10^5^ colony-forming units of MRSA suspension.

The CE was applied topically and was administered twice a day (at 6 am and 6 pm) for 7 days. On days 3 and 7, the area of the wound was measured using a caliper. Subsequently, the rats were anesthetized and euthanized using cervical dislocation. The skin specimens were then collected from the rats and stored in 10% neutral buffer formalin for 24 h.

### Histopathology and immunohistochemistry (IHC)

The skin specimens were processed for histopathology using H and E staining [14], Gram staining [15], and IHC staining against antibody anti-CK-17 (Ks17.E3, catalog number sc-101461; Santa Cruz Biotechnology Inc.). For the CK-17 staining procedure, the following steps were taken. The slides were deparaffinized and dehydrated using xylene and graded alcohol and then rinsed with tap water. Before incubation with the primary antibody, the slides were incubated using a retrieval solution (Bond Epitope Retrieval Solution, catalog number RE7119, Leica Biosystems) at 98°C for 20 min and rinsed with cold water. The slides were then incubated with 4% hydrogen peroxide (Peroxidase Block, catalog number RE7101, Leica Biosystems) for 5 min and rinsed with phosphate buffer saline (PBS). Further, they were incubated with 0.4% casein in PBS (Protein Block, catalog number RE7102, Leica Biosystems) for 5 min and rinsed with PBS. The slides were then incubated with primary antibody anti-CK-17 at 1:5 dilution for 30 min and rinsed using PBS. Rabbit anti-mouse IgG in 10% animal serum (Post-Primary Antibody, catalog number RE7111, Leica Biosystems) was then applied on the slides for 30 min and they were rinsed with PBS. The slides were then incubated with anti-rabbit poly-HRP IgG containing 10% animal serum (Novolink Polymer, RE7112, Leica Biosystems) for 30 min and rinsed using PBS. Following this step, the slides were then incubated with diaminobenzidine chromogen for 5 min, rinsed with tap water, and then counterstained with hematoxylin (catalog number: RE7107, Leica Biosystems) for 30 s. Finally, the slides were rinsed with tap water for 5 min and were subsequently dehydrated, cleared, and mounted. Human breast tissue was used as the positive control for evaluating the IHC of CK-17.

### Morphometry

The data were categorized as macroscopic and microscopic. The macroscopic data were the percentage of the wound area. The microscopic data included epidermal thickness, a score for inflammatory cell infiltration, fibroblast proliferation, percentage of bacterial colonization, and the percentage of expression of CK-17. The histopathological analysis was performed by a single pathologist under blinded conditions. The epidermal thickness, percentage of bacterial colonization, and expression of CK-17 were measured using imaging software (ImageJ, NIH, USA, Public Domain, BSD-2). The inflammatory cell infiltration and fibroblast proliferation were examined using a scoring system.

### Statistical analysis

The numerical data were analyzed using a two-way ANOVA; in case, significant differences were observed, *post hoc* tests were used for pairwise comparisons between the different groups. The categorical data were analyzed using the Kruskal–Wallis and Mann–Whitney *U-* tests. Statistical analyses were performed using SPSS version 16 (IBM Corp., NY, USA) with a probability value at a level of p≤0.05.

## Results

**Figure-1:**
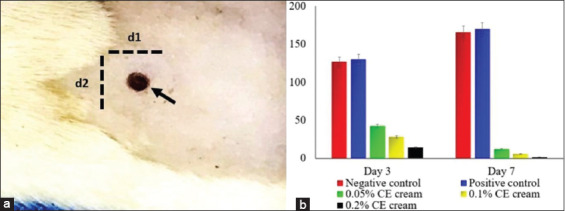
Percentage of wound area. The gross lesion of skin wound infected with methicillin-resistant *Staphylococcus aureus* on the rat’s back (arrow) with the measurement indicator as follows: d1 = diameter 1 and d2 = diameter 2 (a); a trend of percentage of wound area from each group (b).

The group treated with CE cream had a significantly lower percentage of wound area compared to the control group (p=0.001). The control groups showed an increase in the percentage of wound area. All the other groups treated with CE cream groups showed a decrease in the percentage of wound area from day 3 to day 7, where 0.2% CE cream showed the most potent effect on the percentage of wound area compared to the other groups (Figure-1).

**Figure-2:**
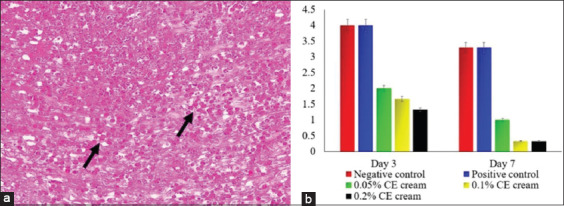
The score of inflammatory cells infiltration of skin wound infected with methicillin-resistant *Staphylococcus aureus*. The neutrophils that massively infiltrated on the wound tissue (arrow) (a); a trend of inflammation’s score from each group (b). H&E staining, 400× (a).

**Figure-3:**
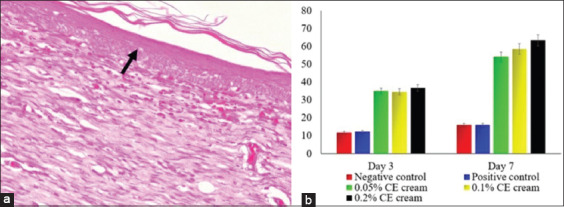
The epidermal thickness of skin wound infected with methicillin-resistant *Staphylococcus aureus*. The measured epidermis during morphometry (arrow) (a); a trend of epidermal thickness from each group (b). H&E staining, 400× (a).

**Figure-4:**
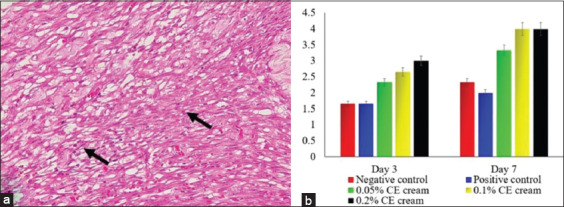
The score of fibroblast proliferation of skin wound infected with methicillin-resistant *Staphylococcus aureus*. The fibroblast that proliferated on the wound tissue (arrow) (a); a trend of fibroblast’s score from each group (b). H&E staining, 400× (a).

Following the healing of the wound, the animals from all the groups that were treated with CE cream demonstrated a decrease in inflammatory cell infiltration, whereas they exhibited an increase in the number of fibroblasts and in the epidermal thickness. The group treated with 0.2% CE cream showed a significant decrease in inflammatory cell infiltration (p≤0.05) (Figure-2) and an increase in epidermal thickness (p≤0.05) (Figure-3) as well as an increased fibroblast proliferation compared to the other groups (p≤0.05) (Figure-4). However, there were no significant differences between the groups treated with 0.05% and 0.1% CE cream regarding the decrease in inflammatory cell infiltration, epidermal thickness, or fibroblast proliferation (p≥0.05).

**Figure-5:**
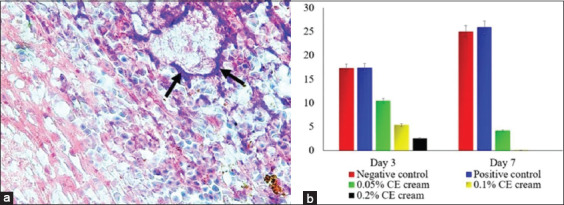
The percentage of bacterial colonization of skin wound infected with methicillin-resistant *Staphylococcus aureus*. The bacterial colonizations were indicated with blue color (arrow) (a); a trend of bacterial colonization from each group (b). Gram staining, 400× (a).

**Figure-6:**
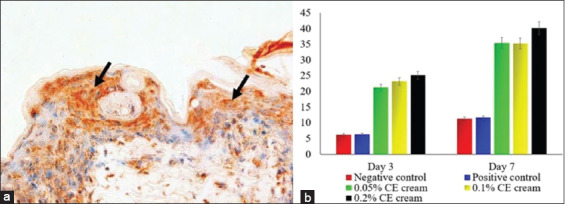
The percentage of cytokeratin (CK)-17 expression on skin wound infected with methicillin-resistant *Staphylococcus aureus*. The high density of CK-17 expression (arrow) is shown by brown color in the suprabasal keratinocytes of wound tissue (A); a trend of CK-17 expression from each group (b). Immunohistochemistry antibody anti-CK-17, diaminobenzidine, 400× (a).

The measurement of the level of bacterial colonization demonstrated the antibacterial effects of CE. Gram staining illustrated that CE cream inhibited bacterial colonization in the wound tissue. About 0.2% CE cream showed the highest antibacterial effects compared to the other groups (p≤0.05) followed by 0.1% and 0.05% CE creams. In contrast, both control groups showed an increase in bacterial colonization in the wound area (Figure-5). The inhibition of bacterial colonization in wounds promotes skin reepithelialization that is not only associated with an increase in epidermal thickness but also an upregulation of CK-17 expression among the keratinocytes. CK-17 expression increased in all groups from day 3 to 7 during the study period. However, the control group showed a significantly lower CK-17 density compared to the CE-treated groups (p=0.001) (p≤0.05) (Figure-6). This study demonstrated that topical application of CE cream affected CK-17 expression in keratinocytes. Treatment with 0.2% CE cream appeared to be the most effective in increasing CK-17 expression, compared to other CE-treated groups (p≤0.05). However, treatment with 0.1% and 0.05% CE creams did not show any significant differences from each other (Figure-6).

## Discussion

CE contains several bioactive components that have potential antioxidant as well as antibacterial properties. This study demonstrates that CE inhibits the growth of MRSA *in vitro* at 0.1% concentration. Phenolic compounds are one of the most prominent components of herbal extracts; this includes CE. Phenolic compounds are potential antibacterial agents [16]. They act by interacting with the bacterial membrane containing peptidoglycans [17]. This phenolic-peptidoglycan binding causes changes in the membrane rigidity and permeability [18]. Further, a study has reported that the utilization of an herbal antioxidant from *Stephania suberosa*, which contains alkaloids, could inhibit the synthesis of peptidoglycans and also inhibit β-lactamase, resulting in the impairment of membrane permeability [19]. These mechanisms affect the ability of MRSA to receive low osmotic and high ionic pressure, thus impairing bacterial colonization *in vitro*.

In the *in vivo* study, the potential effects of CE cream were demonstrated by a decrease in bacterial colonization within the wound matrix. The decrease in bacterial colonization in the tissue generates minimal leukocyte infiltration and minimal oxidative stress. Further, the CE cream contains exogenous antioxidants that promote the oxidant scavenging activity in the wound tissue and increases both cellular and immune responses in wound healing. Antioxidants such as flavonoids, phenolics, alkaloids, sterols, and tannins may influence the endogenous antioxidant levels in cells of wound tissue [20]. Increasing levels of endogenous antioxidants in living cells promote their ability to regenerate, infiltrate, and proliferate during the healing process [21]. In addition, the synergistic effects of radical scavenging activity and increasing levels of endogenous antioxidants decrease levels of ROS in wound tissue. These mechanisms were demonstrated in this study which showed that there was minimum inflammatory cell infiltration compared to the controls.

Moreover, the antioxidant components of CE can function as potential anti-inflammatory agents due to their ability to inhibit leukocyte adhesion in the wound area [22]. The inhibition of leukocyte adhesion decreases the expression of pain receptors through the cyclooxygenase route. Even a brief inflammatory period increases fibroblast and keratinocyte activity, and the healing mechanism is expedited [23]. On the wound tissue, keratinocytes induce reepithelialization through their migration from the wound margin and underlying dermis [24,25]. However, before the keratinocyte migration, the CK genes are expressed among the epidermal keratinocytes, one of them being CK-17 [10]. Keratinocytes induce a high degree of expression of CK-17 within an hour after injury and this continues to increase until they cover the entire wound bed by the 6^th^ day of acute wound healing [26]. The utilization of antioxidants can induce an increased CK-17 expression within the wound tissue. CK-17 activation promotes the pseudopodia of keratinocytes during their migration [27]. A previous study reported that the impairment of CK-17 expression in the wound area significantly inhibits wound healing [28]. In addition, CK-17 promotes not only reepithelialization but also matrix granulation and fibroblast activity. This study demonstrated that CE cream promotes reepithelialization and keratinocyte activation through CK-17 expression in the suprabasal region of the epidermis and dermis. In this study, all the molecular and cellular processes that were involved in wound healing could also be identified macroscopically by a decrease in the wound area.

## Conclusion

Celery (*A. graveolens*) extract has a beneficial effect as an antibacterial and is also a source of antioxidants. It also enhances promoters of wound healing. These effects were demonstrated by the inhibition of MRSA colonization both *in vitro* and *in vivo*, decreasing inflammatory cell infiltration, and increasing the activity of dermal keratinocytes in the expression of CK-17. The increased expression of CK-17 in the wound bed promotes fibroblast proliferation and reepithelialization.

## Authors’ Contributions

YAP, CSR, AR, MS, and DW designed the study and drafting the manuscript. YAP, CSR, and DW performed all the experimental procedures. AR and MS formulated the extract and cream. YAP and CSR conducted data analysis and interpretation. All authors read and approved on the final version of the manuscript.

## Data Availability

The data used to support the findings of this study are available from the corresponding author upon request.

## Implications

The sub-population of keratinocytes that expressed CK-17 in the wound bed is not a single homogenous cell population, and there may be many more CK types that influence wound healing. Further study is necessary to evaluate if there is r involvement of other CK subtypes in wound healing after CE cream therapy.
